# HIV-1 Gag Forms Ribonucleoprotein Complexes with Unspliced Viral RNA at Transcription Sites

**DOI:** 10.3390/v12111281

**Published:** 2020-11-09

**Authors:** Kevin M. Tuffy, Rebecca J. Kaddis Maldonado, Jordan Chang, Paul Rosenfeld, Alan Cochrane, Leslie J. Parent

**Affiliations:** 1 Division of Infectious Diseases and Epidemiology, Department of Medicine, Penn State College of Medicine, Hershey, PA 17033, USA; tuffy.kevin@yahoo.com (K.M.T.); rkaddis@pennstatehealth.psu.edu (R.J.K.M.); jchang3@pennstatehealth.psu.edu (J.C.); 2 The Institute of Medical Sciences, University of Toronto, Toronto, ON MS5 1A8, Canada; paul.rosenfeld@utoronto.ca (P.R.); alan.cochrane@utoronto.ca (A.C.); 3 Department of Molecular Genetics, University of Toronto, Toronto, ON MS5 1A8, Canada; 4 Department of Microbiology and Immunology, Penn State College of Medicine, Hershey, PA 17033, USA

**Keywords:** HIV-1 Gag, viral RNA, nucleus, Rev

## Abstract

The ability of the retroviral Gag protein of Rous sarcoma virus (RSV) to transiently traffic through the nucleus is well-established and has been implicated in genomic RNA (gRNA) packaging Although other retroviral Gag proteins (human immunodeficiency virus type 1, HIV-1; feline immunodeficiency virus, FIV; Mason-Pfizer monkey virus, MPMV; mouse mammary tumor virus, MMTV; murine leukemia virus, MLV; and prototype foamy virus, PFV) have also been observed in the nucleus, little is known about what, if any, role nuclear trafficking plays in those viruses. In the case of HIV-1, the Gag protein interacts in nucleoli with the regulatory protein Rev, which facilitates nuclear export of gRNA. Based on the knowledge that RSV Gag forms viral ribonucleoprotein (RNPs) complexes with unspliced viral RNA (USvRNA) in the nucleus, we hypothesized that the interaction of HIV-1 Gag with Rev could be mediated through vRNA to form HIV-1 RNPs. Using inducible HIV-1 proviral constructs, we visualized HIV-1 Gag and USvRNA in discrete foci in the nuclei of HeLa cells by confocal microscopy. Two-dimensional co-localization and RNA-immunoprecipitation of fractionated cells revealed that interaction of nuclear HIV-1 Gag with USvRNA was specific. Interestingly, treatment of cells with transcription inhibitors reduced the number of HIV-1 Gag and USvRNA nuclear foci, yet resulted in an increase in the degree of Gag co-localization with USvRNA, suggesting that Gag accumulates on newly synthesized viral transcripts. Three-dimensional imaging analysis revealed that HIV-1 Gag localized to the perichromatin space and associated with USvRNA and Rev in a tripartite RNP complex. To examine a more biologically relevant cell, latently infected CD4+ T cells were treated with prostratin to stimulate NF-κB mediated transcription, demonstrating striking localization of full-length Gag at HIV-1 transcriptional burst site, which was labelled with USvRNA-specific riboprobes. In addition, smaller HIV-1 RNPs were observed in the nuclei of these cells. These data suggest that HIV-1 Gag binds to unspliced viral transcripts produced at the proviral integration site, forming vRNPs in the nucleus.

## 1. Introduction

Retroviruses replicate their RNA genomes through a DNA intermediate that is integrated into the host cell chromosome. This DNA provirus is transcribed by cellular machinery into viral RNA (vRNA) transcripts, which can be spliced into subgenomic messenger RNAs (mRNA) that encode for a variety of viral proteins [1]. Alternatively, the vRNA can remain unspliced (US) and function as a template for Gag and Gag-Pol translation, or as the viral genome, that is packaged into nascent virions. The Gag polyprotein selects viral genomic RNA (gRNA) for encapsidation into virions through an interaction between the RNA binding motif located in the nucleocapsid (NC) domain of Gag and the psi packaging signal (Ψ) in the 5′ untranslated region (5′UTR) of the viral gRNA [2,3]. The initial Gag-gRNA interaction that forms the vRNP complex has been thought to occur in the cytoplasm or at the plasma membrane where budding ensues [4,5,6,7,8]. However, this traditional assembly model was challenged by the discovery that the RSV Gag protein transiently localizes to the nucleus, a requirement for efficient gRNA packaging [9,10].

RSV Gag contains nuclear localization signals in the MA (matrix) and NC (nucleocapsid) domains and a CRM1-dependent nuclear export signal in p10 [11,12,13,14,15]. Formation of the RSV vRNP complex facilitates binding to the CRM1-Ran GTP export complex, suggesting that directionality of nucleocytoplasmic transport is mediated by Gag-nucleic acid binding [11]. RSV Gag mutants exhibiting decreased nuclear trafficking produce viral particles that have reduced levels of gRNA encapsidation, and restoration of nuclear trafficking through the addition of an exogenous NES reconstitutes gRNA packaging [9]. Moreover, live-cell confocal imaging and biophysical experiments revealed that RSV Gag binds USvRNA at transcriptional bursts, and the nascent viral RNP traffics into the cytoplasm [16]. Together these data suggest that RSV Gag interacts with gRNA co-transcriptionally to form an export competent complex that exits the nucleus in a CRM1-dependent fashion, which may ultimately be encapsidated during the budding process [9,11,12,13,14,15,16,17].

The Gag proteins of several other retroviruses have been observed in the nucleus, including human immunodeficiency virus type-1 (HIV-1) [18,19], feline immunodeficiency virus (FIV) [20], Mason-Pfizer monkey virus (MPMV) [21,22], mouse mammary tumor virus (MMTV) [23], murine leukemia virus (MLV) [24], and prototype foamy virus, PFV [25]. However, the nuclear function of these retroviral Gag proteins is incompletely understood. For example, the mechanisms by which HIV-1 Gag is imported and exported from the nucleus remain unknown. HIV-1 Gag nuclear export does not appear to be mediated by CRM1, as treatment of Gag-expressing cells with leptomycin B (LMB), a CRM1 inhibitor, does not result in dramatic nuclear Gag accumulation [21]. In addition, a subpopulation of HIV-1 Gag localizes to nucleoli where it was shown via Förster resonance energy transfer (FRET) to associate with the HIV-1 regulatory protein Rev, which exports partially spliced and USvRNA from the nucleus [19]. It is possible that an interaction between HIV-1 Gag and Rev in the nucleus may be mediated by vRNA. Therefore, we sought to investigate whether HIV-1 Gag associates with vRNA and Rev in the nucleus.

Utilizing a cell line that contains a stably integrated, doxycycline (dox)-inducible HIV-1 provirus expressing Gag fused to GFP (HeLa HIV.Gag-GFP rtTA), we used confocal microscopy to demonstrate HIV-1 Gag specifically co-localizes with USvRNA in the nucleus in a transcription-dependent fashion. Additionally, RNA-immunoprecipitation of Gag-vRNA complexes from fractionated cells exhibited an enrichment of USvRNA with HIV-1 Gag in both the nucleus and cytoplasm. Furthermore, we visualized three-dimensional co-localization between HIV-1 Gag, USvRNA, and Rev, suggesting that the previously reported Gag-Rev association may be mediated by vRNA. Finally, in the CD4+ T cell line JLat 10.6 induced to express the latent provirus, Gag trafficked to the transcriptional burst site and co-localized with USvRNA, suggesting that HIV-1 RNP complexes form in the nucleus of a biologically relevant cell line. Taken together, these data propose that HIV-1 Gag associates with USvRNA co-transcriptionally to form nuclear vRNP complexes.

## 2. Materials and Methods

### 2.1. Plasmids, Cell Culture, and Transfections

HeLa HIV.Gag-GFP rtTA (rtTA, tetracycline transactivator protein) is a HeLa cell line containing a stably integrated dox-inducible HIV-1 provirus, as previously described [19,26]. The modified LTRs contain a mutated TAR (*trans*-activation response element), two NF-κB sites, eight copies of the tetO sites in the promoter, and three SP1 sites [27,28,29]. To allow visualization of Gag, this cell line expresses HIV-1 Gag-GFP by replacing a portion of Pol with an in-frame fusion of GFP to Gag. HIV-1 proviral expression occurs upon the addition of doxycycline to the cell medium, and the expression of Gag is dependent on HIV-1 Rev. Additionally, the HeLa HIV.Gag-GFP rtTA cell line was further modified to express a dox-inducible Rev-BFP fusion protein from a separate transgene inserted into the host cell genome utilizing *piggyBac* transposon mediated gene transfer [30,31] to generate a HIV-1 Gag-GFP rtTA Rev-BFP cell line. The inducible Rev-BFP was created by PCR amplifying BFP flanked by AgeI and NotI sites and replacing YFP in pRev-YFP [23] through standard restriction enzyme cloning. Rev-BFP was then excised with NheI and NotI and inserted into PB-T-PAF (a gift from Dr. James Rini, University of Toronto) [31], a dox-inducible *piggyBac* transposon vector. Following transfection of HIV-1 Gag-GFP rtTA cell with PB-T-PAF Rev-BFP and PBase, *piggyBac* transposase (Sanger Institute), positive clones were selected for by puromycin resistance [31]. Both HeLa HIV.Gag-GFP rtTA and HeLa HIV.Gag-GFP rtTA Rev-BFP cells were maintained as previously described [19,32].

A separate HIV Gag-CFP rtTA proviral plasmid (pHIV.Gag-CFP rtTA) was constructed using pNL4-3 24x MS2-MBL as starting material (a gift from Dr. Nathan Sherer, University of Wisconsin-Madison [33]) to create an inpendent dox-inducinble HIV-1 provirus expressing Gag = CFPs. A 1.9 kB MscI fragment was excised from *pol*. Three copies of MS2 stem loops sit between *gag-cfp* and the truncated *pol* gene. The 5′ and 3′ long terminal repeats (LTR) were replaced with a dox-inducible promoter (TRE-tetracycline responsive element). The TRE was PCR amplified from the plasmid pHIV-1 rtTA Gag ZIP construct (provided by Alan Cochrane) using primers 5′-ATG CGA CGT CTG GAA GGG CTA ATT CAC TCC CAA CGA AGA CAA GAT-3′ and 5′-CGA TGC GCG CTT CAG CAA GCC GAG TCC TGC GTC GAG AGA TCT CCT CTG GCT TTA CTT-3′ containing AatII and BssHI cut sites for the 5′LTR and primers 5′-AAT TCT CGA GGG CTC TGG TAG TTG GAA GGG CTA ATT CAC TC-3′ and 5′-GCA TGC CGG CGC GCC ACT GCT AGA GAT T-3′ containing XhoI and NaeI cut sites for the 3′ LTR. Additionally, the original luciferase gene in pNL4-3 was replaced with rtTA from the HIV-1 rtTA Gag ZIP that was amplified with primers 5′-AAG GTT TGC GGC CGC ATG TCT AGA CTG GAC AAG AGC-3′ and 5′-AAG GTT CTC GAG GCT AGC ACT TAG TTA CCC GGG GAG CAT GTC-3′ and restriction cloned into NotI and XhoI sites. The resulting pHIV. Gag-CFP rtTA plasmid was further modified with the replacement of the CFP fluorophore tag to SNAP-tag using PmeI and SacII cut sites (pHIV.Gag-SNAP-tag rtTA). SNAP-tag PCR amplification was achieved using primers 5′-GCA TGT TTA AAC GGA CCG GTG ACA AAA CTG CGA AAT GAA GCG-3′ and 5′-CAG TCC GCG GTT AAC CCA GCC CAG GCT TGC-3′.

A HeLa cell line containing a stably-integrated rtTA (HeLa rtTA) was generated by co-transfecting 3 μg pPB-t-rtTA with 1.2 μL Super Piggybac Transposase (System Biosciences, Palo Alto, CA, USA, PB210PA-1) followed by selection of rtTA expressing cells with 8 μg/mL blasticidin. The HeLa cells expressing rtTA were utilized in experiments with doc-inducible constructs pHIV.Gag-CFP rtTA and pHIV.Gag-SNAP rtTA.

Plasmids expressing Rev-independent pHIV Gag-CFP/GFP [19,34], Rev-dependent pHIV Gag-CFP (a gift from Dr. Eric Poeschla, University of Colorado School of Medicine) [35], pRev-YFP [23], and pEGFP-C1 (Takara Bio USA, Mountain View, CA, USA) were previously described. Human cervical cancer (HeLa) cells were maintained as previously described [19,32]. Human embryonic kidney (293T) cells were grown in Dulbecco’s modified Eagle medium with 10% fetal bovine serum, 1% sodium pyruvate, pen/strep, and fungizone. 293T cells were transfected using calcium phosphate [36], whereas transfections of HeLa cells were performed using Lipofectamine 2000 (Thermo Fisher Scientific-Invitrogen) according to manufactures’ standard protocol. pGem was used to normalize all Lipofectamine 2000 transfections to 4 µg of total DNA per 10 µL of Lipofectamine 2000 reagent. Transfections were conducted for 16–18 h for all experiments.

Human osteosarcoma (U2OS) cells were maintained similarly as HeLa cells using Dulbecco’s modified Eagle medium with 10% fetal bovine serum, pen/strep, and fungizone. Transfection of U2OS cells with HIV-1 Gag-CFP rtTA were conducted with Lipofectamine 2000 (Thermo Fisher Scientific-Invitrogen) as described above. Conversely, transfection of HeLa cells with HIV-1 Gag-SNAP-tag rtTA were performed using jetOPTIMUS DNA Transfection Reagent (Polyplus transfection, New York, NY, USA) to 2 µg of total DNA per 1 µL jetOPTIMUS reagent. For these experiments, additional rtTA expressed from pB-rtTA (a gift from Jim Rini, University of Toronto) was added in *trans*.

JLat cells clone 10.6 (from Eric Verdin, obtained through the NIH AIDS Reagent Program, Division of AIDS, NIAID, NIH, Germantown, MD, USA) were grown in RPMI medium supplemented with 10% fetal bovine serum and pen/strep. JLat cells are Jurket derived T cells that contain a latent HIV-1 provirus in which *nef* was replaced with *gfp* and a frameshift mutation was made to prevent *env* expression [37]. JLat 10.6 cells contain integrated proviruses that can be reactivated from latency by treatment with prostratin, which activates NF-κB sites in the 5′UTR to induce HIV-1 expression.

### 2.2. Dox-Induction and Visualization of USvRNA and GAPDH RNA in HeLa and U2OS Cells

HIV-1 provirus expression was induced in HeLa HIV.Gag-GFP rtTA and HeLa HIV.Gag-GFP rtTA Rev-BFP cells grown on 1.5 mm cover slips by the addition of dox (2 µg/mL) to the cell media for 24 h. Following dox induction, HeLa HIV.Gag-GFP rtTA and HeLa HIV.Gag-GFP rtTA Rev-BFP cells were fixed in 3.7% formaldehyde in PBS for 10 min at room temperature, permeabilized in 70% EtOH at 4 °C for a minimum of 1 h, and rehydrated in wash buffer (10% formamide in 2X SSPE) for 20 min. Next, hybridization buffer (10% dextran sulfate, 2X SSPE, 10% formamide) containing a mix of either 48 different Stellaris^®^ single molecule fluorescence in situ hybridization (smFISH) probes conjugated to Quasar 570 (Biosearch Technologies, Novato, CA, USA) targeting the *gag* coding region or 80 Stellaris^®^ smFISH probes targeting human GAPDH transcripts, were added to the rehydrated cells and incubated overnight at 37 °C in a sealed humid chamber. Following the incubation, cells were washed in warm wash buffer for 30 min at 37 °C and then incubated for an additional 30 min at 37 °C in wash buffer containing 5 µg/mL DAPI before being mounted onto slides. The same dox-induction protocol was applied to U2OS and HeLa cells transfected with pHIV.Gag-CFP rtTA or pHIV.Gag-SNAP-tag rtTA.

For cells not subjected to FISH, they were fixed with 3.7% paraformaldehyde and 0.4M KOH in PHEM buffer [3.6% piperazine-N,N = -bis(2-ethanesulfonic acid) (PIPES), 1.3% HEPES, 0.76% EGTA, 0.198% MgSO4, pH to 7.0 with 10 M KOH] [38] for 15 min at room temperature with gentle rocking. Following fixation, cells were then washed in PBS and DAPI stained before mounting onto slides using ProLong Diamond Antifade Mountant (Thermo Fisher Scientific-Invitrogen).

For cells transfected with pHIV.Gag-SNAP-tag rtTA, cells were incubated with 50 nM SNAP-tag ligand Janelia Fluor 549 (JF549) [39] at 37 °C for 1 h. Cells were washed with warm standard buffer and fixed with fixation media prior to DAPI staining and mounting.

During dox-induction, HeLa HIV.Gag-GFP rtTA cells were treated with either 150 µM of 5,6-Dichloro-1-β-D-ribofuranosylbenzimidazole (DRB) or 4 µg/mL Actinomycin D (Act D) in cell media to inhibit transcription for 30 or 60 min prior to fixation (i.e., 30 min treatment corresponds to drug being added for last 30 min of the 24 h induction). In separate experiments, HeLa HIV.Gag-GFP rtTA cells were treated with 20 nM of the CRM1 inhibitor LMB for 30, 60, 90, or 120 min prior to fixation.

### 2.3. Prostratin Induction and Sequential IF/FISH Labeling of USvRNA and Gag in JLat 10.6 Cells

JLat 10.6 cells were induced with 1 µg/mL prostratin for 24 h. Cells were washed with PBS, then resuspended in PBS and allowed to attached to a glass coverslip for 30 min at 37 °C before they were fixed with 3.7% formaldehyde in 1× PBS for 10 min [40]. Sequential IF/smFISH was performed using a modified Stellaris^®^ RNA FISH Protocol for Sequential IF + FISH in Adherent Cells (Biosearch Technologies, Novato, CA, USA); [41,42]). Following fixation, cells were washed twice with 1× PBS then permeabilized in 0.1% Triton X-100 in 1× PBS for 5 min before being washed again in 1× PBS. Gag was detected using anti-p24 antibody diluted in 1× PBS and applied to cells for 1 h at room temperature. Cells were washed three times in 1× PBS for 10 min before being incubated in secondary antibody in 1× PBS for 1 h at room temperature. Cells were washed again three times in 1× PBS for 10 min. Following washing, cells were subjected to a second fixation in 3.7% formaldehyde in 1× PBS for 10 min at room temperature and then washed twice in 1× PBS. smFISH to detect USvRNA was performed the same as previously mentioned.

To evaluate whether full-length Gag was present in JLat 10.6 cells, cells either remained untreated or were induced with prostratin as above. Cells were lysed in RIPA buffer (50 mM Tris-HCl pH 7.2, 150 mM NaCl, 1% Triton ×100, 0.01% DOC, 0.1% SDS) and subjected to immunoblot analysis using the Stain-Free Imaging Technology (BioRad, Hercules, CA, USA). Following transfer to PVDF membrane and blocking 1 h in 5% milk. 1× TBST Gag was detected using purified mouse anti-p24 supernatant from anti-HIV-1 Gag hybridoma 183 (NIH AIDS Reagent Program, Division of AIDS, NIAID, NIH, Germantown, MD, USA) at a dilution of 1:300 for 1 h and GAPDH was detected with rabbit polyclonal antibody to GAPDH ( MilliporeSigma, St. Louis, MO, USA) at 1:5000 dilution. Appropriate secondary HRP-conjugated antibody at 1:5000 (Jackson ImmunoResearch, West Grove, PA, USA; rabbit Cat. #715-036-150 and mouse Cat. #711-036-152) and visualized with Clarity Western ECL Substrate (BioRadCat. #1705060) using the ChemiDoc MP Imager (BioRad).

### 2.4. Confocal Microscopy

Following fixation and processing, cells were subjected to confocal microscopy using a Leica SP8 (Leica Microsystems, Buffalo Grove, IL, USA) confocal microscope equipped with a White Light Laser using a 63X/NA1.4 oil immersion objective. Single fluorophore controls were used to ensure that no crosstalk occurred. Sequential scanning of each channel was utilized with a frame average of four and an imaging speed of 400 Hz. Images were subjected to Guassian filters and histogram adjustments for display purposes, and three-dimensional surface renderings of Z-stacks, cross-sections, and co-localization channels were generated in Imaris 9.3 image analysis software (Bitplane Inc., Concord, MA, USA).

### 2.5. Quantitative Image Analysis

Fluorescent focus detection and two-dimensional co-localization analysis were performed using DIPimage (TU Delft, Delft University of Technology, Delft, The Netherlands) and custom MATLAB (The MathWorks, Inc., Natick, MA, USA) scripts created by Dr. Stephen Lockett, Director of Optical Microscopy and Analysis Laboratory at National Cancer Institute in Fredrick, MD [16,43]. Fluorescent foci were detected with a minimum intensity threshold of 20–75 for each image. A focus detected in one channel that was within 250 nm of a focus detected in the other channel was considered co-localized based on the resolution limit of our confocal microscope. The percentage of co-localized foci in each channel for individual images was determined and the mean ± standard error of the mean was calculated. Two-tailed t-tests were used to compare untreated controls to the appropriate drug treatment.

Three-dimensional co-localization between HIV-1 Gag, USvRNA, and Rev was performed in Imaris 9.3 (Bitplane Inc., Concord, MA, USA) by generating surface renderings from confocal Z-stacks of induced HIV-1 Gag-GFP rtTA Rev-BFP cells that contained nuclear Gag-GFP foci and a region of interest was produced by masking the Rev-BFP channel. A co-localization channel between HIV-1 Gag-GFP and USvRNA was generated using the same manual threshold levels for every cell in the dataset, within the Rev-BFP mask [44].

### 2.6. Subcellular Fractionations

Following dox-induction, HeLa HIV.Gag-GFP rtTA cells or HeLa rtTA cells transfected with either pHIV.Gag-GFP rtTA or pHIV.Gag-SNAP-tag rtTA were separated into cytoplasmic and nuclear fractions by a modification to a previously described protocol [45]. Briefly, cells were washed three times in cold 1× PBS followed by the addition of cytoplasmic lysis buffer (10 mM Hepes pH7.9, 10 mM KCl, 0.1 mM EDTA, 0.4% [vol/vol] Nodiet P40, 1 mM DTT, and Roche protease inhibitor cOmplete tablets EDTA free to 1×) to the monolayer and collected by gentle scraping. For RNA-immunoprecipitation experiments cytoplasmic buffer was supplemented with SUPERase-in [1 µL/mL]. The lysis reaction was incubated on ice for 10 min, then spun down at 16,000× *g* at 4 °C for 10 min, and the supernatant was removed as cytoplasmic fraction. The pellet was washed three times in cytoplasmic lysis buffer, then resuspended in nuclear extraction buffer (20 mM Hepes pH 7.9, 0.4 M NaCl, 1 mM EDTA, 1 mM DTT, Roche protease inhibitor cOmplete tablets EDTA free (MilliporeSigma, St. Louis, MO, USA) to 1×), and rotated end-over-end for 15 min at 4 °C. Minor modifications were made to the nuclear extraction buffer for fractions of HIV-1 Gag transfected cells (with 10 mM NaCl). For RNA-immunoprecipitation experiments nuclear extraction buffer was supplemented with SUPERase-in (Thermo Fisher Scientific-Invitrogen, 1 µL/mL). Samples were spun for 10 min at 16,000× *g* at 4 °C and supernatant was removed as nuclear fraction. The same method was utilized in HeLa cells transfected with either Rev-independent pHIV-1 Gag-GFP or pEGFP-C1 except following collection of the nuclear fraction, the remaining nuclear pellet was resuspended in SDS loading buffer (125 mM Tris-HCl, pH 6.8, 20% glycerol, 0.5% bromophenol blue, 4% SDS, and 10% β-mercaptoethanol), boiled for 10 min, and centrifuged at 16,000× *g*. Supernatant was removed as nuclear pellet fraction. 50 µg of total protein from the cytoplasmic and nuclear fractions along with 25 µL nuclear pellet fraction were run on an SDS-PAGE gel. Fractionation of HeLa rtTA cells transfected with pHIV.Gag-CFP rtTA, pHIV.Gag-SNAP-tag rtTA, or pECFP-N1 was performed similarly as mentioned above. Instead, 10 μg of total protein from the cytoplasmic and nuclear fraction was loaded on an SDS-PAGE gel. Gels were transferred onto PVDF and immunoblotted with mouse monoclonal anti-HIV-1 p24 (24-3 or 24-44) (NIH AIDS reagents Program, Division of AIDS, NIAID, NIH, Germantown, MD, USA, 6521), rabbit polyclonal anti-GFP (AbCam, Cambridge, MA, USA ab290), rabbit polyclonal anti-Calnexin (Stressgen, Enzo Life Sciences, Inc., Farmingdale, NY, USA, SPA-865), rabbit monoclonal anit-MED4 (AbCam, Cambridge, MA, USA, ab129170), mouse monoclonal anti-fibrillarin (AbCam, ab4566) followed by secondary anti-rabbit or anti-mouse immunoglobulin G conjugated to horseradish peroxidase (MilliporeSigma) and detected using chemiluminescence.

Additionally, subcellular chromatin fractionations were conducted following a previously described protocol [46] with minor modifications. Following transfection with either Rev-independent pHIV Gag-CFP or Rev-dependent pHIV Gag-CFP and pRev-YFP, 293T cells were collected, washed twice in PBS, and resuspended in cold TM2 buffer (10 mM Tris-HCl pH 7.4, 2 mM MgCl_2_) by gentle vortexing. Cells were incubated for 1 min on ice prior to the addition of Nonidet P40 to 1.5% (vol/vol), vortexed and incubated for an additional 5 min. Lysate was then pelleted at 5000× *g* for 10 min at 4 °C. Supernatant was discarded and pellet was resuspended in warm TM2 buffer with CaCl_2_ added to 1 mM and Omnicleave (Epicentre-Lucigen Corporation, Middleton, WI, USA) to 1.6 U/mL and incubated for 10 min at 37 °C. Reaction was then stopped by the addition EGTA to 2 mM on ice. Samples were the pelleted at 5000× *g* for 10 min at 4 °C, supernatant was discarded and pellet was washed in of TM2 buffer. The pellet was then resuspended in 150 mM buffer (10 mM Tris-HCl pH 7.4, 2 mM MgCl_2_, 150 mM NaCl, 2 mM EGTA, 100 mM, 10% (vol/vol) Triton ×100) and rotated end-over-end for 2 h at 4 °C. Samples were centrifuged at 5000× *g* for 10 min at 4 °C and the supernatant was removed as the chromatin 150 mM fraction (Chr 150). The remaining pellet was resuspended in 600 mM (10 mM Tris-HCl pH 7.4, 2 mM MgCl_2_, 600 mM NaCl, 2 mM EGTA, 100 mM, 10% (vol/vol) Triton ×100) and rotated end-over-end overnight at 4 °C. The next day samples are pelleted at 3000× *g* for 10 min at 4 °C, and the supernatant was removed as the chromatin 600 mM fraction (Chr 600). Whole cell lysates (WCL) were prepared by collecting cells from each transfection, incubating on ice for 30 min in RIPA buffer (50 mM Tris-HCl pH 7.2, 150 mM NaCl, 1% Triton ×100, 0.01% DOC, 0.1% SDS), and subsequently centrifuged at 16,000× *g* for 30 min at 4 °C. Following Bradford assay to determine protein concentration, 50 µg of total protein from the WCL and equal volumes of the Chr 150 and Chr 600 fractions were loaded on the gel, transferred onto PVDF and immunoblotted with rabbit polyclonal anti-GFP (AbCam, Cambridge, MA, USA, ab290), mouse monoclonal anti-GAPDH (UBPBio, Ubiquitin-Proteasome Biotechnologies, LLC, Aurora, CO, USA, Y1040), mouse monoclonal anit-RCC1 (AbCam, ab54600), and mouse monoclonal anti-Histone H2B (AbCam, ab52484) followed by secondary anti-rabbit or anti-mouse immunoglobulin G conjugated to horseradish peroxidase (MilliporeSigma) and detected using chemiluminescence.

### 2.7. RNA-Immunoprecipitation and Biochemical Analysis

Mouse anti-GFP (Roche, MilliporeSigma, Cat.11814460001) or control Mouse IgG (Jackson ImmunoResearch, West Grove, PA, USA, Cat.015-000-003) were pre-ligated to magnetic bead suspension (Bio-Rad Sure Beads Protein G, 10 µg/µL ≥ 6 µg/mg binding capacity, BioRad). Beads were washed in three times in 3× sterile filtered PBS-T (0.1% Tween-20) then incubated by rotating overnight at 4 °C with the appropriate cytoplasmic or nuclear fractions. IP complexes were isolated by magnetic separator and the supernatants reserved for downstream analysis. Beads were washed with nuclear extraction buffer and transferred to a new tube followed by an additional nuclear extraction buffer wash and two washes with lysis buffer. IP suspensions were separated into two equal volumes then resuspended in cytoplasmic lysis buffer for immunoblot analysis and TRIzol reagent (Invitrogen Cat. 15596026) for RNA analysis. Harvested RNA was resuspended in water and an aliquot was reverse transcribed to cDNA with M-MLV Reverse Transcriptase (Thermo Fisher Scientific-Invitrogen Cat. 28025013). Diluted cDNAs were used to quantitate RNA targets: US (forward 5′-CTG AAG CGC GCA CGG CAA -3′, reverse 5′-GAC GCT CTC GCA CCC ATC TC-3′), SS (forward 5′-GGC GGC GAC TGG AAG AAG C-3′, reverse 5′-CTA TGA TTA CTA TGG ACC ACA C-3′), MS (forward 5′-GAC TCA TCA AGT TTC TCT ATC AAA-3′, reverse 5′-AGT CTC TCA AGC GGT GGT-3′), and β-actin (forward 5′-GAG CGG TTC CGC TGC CCT GAG GCA CTC-3′, reverse 5′-GGG CAG TGA TCT CCT TCT GCA TCC TG-3′) as previously described [26]. Fold enrichments over 10% input control were calculated using the 2^−∆∆Ct^ method [47]. Lysate and IP samples were eluted in boiling dissociation buffer (250 mM Tris-HCL pH6.8, 5% SDS, 50% glycerol, 0.1% bromophenol blue, and 5% β-mercaptoethanol) and supernatants were run on an SDS-PAGE gel. The gel was transferred onto PVDF and immunoblotted with mouse anti-p24 HIV-1 Gag hybridoma 183 (NIH AIDS Reagent Program, Division of AIDS, NIAID, NIH, Germantown, MD, USA). Blots of nuclear and cytoplasmic lysates were incubated with mouse anti-Lamin A/C (BD Transduction Laboratories, Cat. 612162), and mouse anti-α-Tubulin ((MilliporeSigma, Cat. T9026). All blots were incubated with anti-mouse secondary immunoglobulin G conjugated to horseradish peroxidase (Jackson ImmunoResearch, Cat. 711-036-152) and detected using chemiluminescence.

## 3. Results

### 3.1. HIV-1 Gag Forms Nuclear Foci in Multiple Cell Types

**Figure 1:**
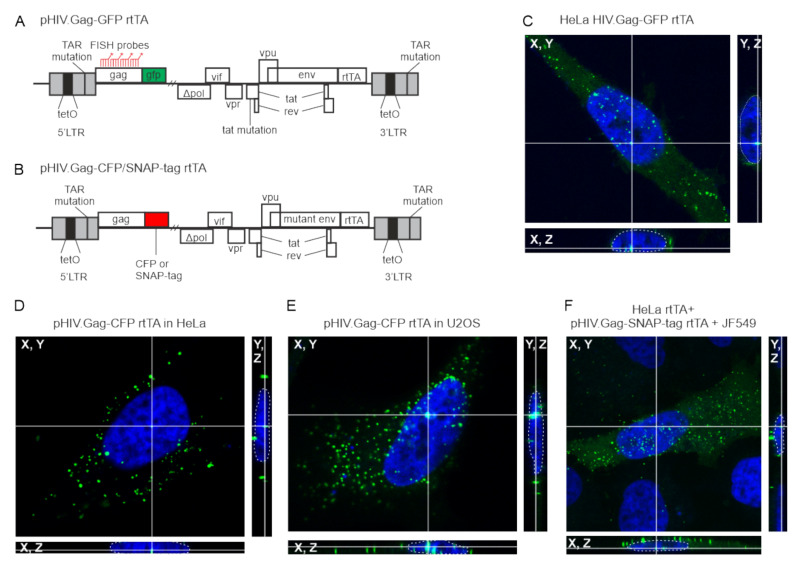
HIV-1 Gag foci are present in the nucleus of HeLa and U2OS cells regardless of the nature of the tag fused to the C-terminus of Gag, which was expressed in a Rev-dependent fashion using the constructs shown in panels A and B. (**A**) Schematic of doxycycline-inducible HIV.Gag-GFP rtTA proviral construct expressing Gag fused to GFP, which is stably integrated in HeLa cells (HeLa HIV.Gag-GFP rtTA). The 5′LTR contains a mutated TAR, two NF-κB sites, eight copies of the tetO sites in the promoter, and three SP1 sites. (**B**) Schematic of pHIV.Gag-CFP/SNAP-tag rtTA doxycycline-inducible construct with the same 5′UTR sequences described in panel A, which expresses Gag fused to either CFP or SNAP-tag. (**C**) HeLa HIV.Gag-GFP rtTA cell line contains the stably-integrated proviral construct shown in panel A. Numerous Gag foci (green) were seen in the nucleus, which was stained with DAPI (blue). Three-dimensional reconstruction of a confocal z-series is shown with crosshairs through a single Gag focus to demonstrate that Gag was present inside the nucleus (outlined by a dashed white line) in all three planes. (**D**) HeLa cells co-expressing pHIV.Gag-CFP rtTA with pPB-t-rtTA. (**E**) U2OS cells transfected with pHIV.Gag-CFP rtTA and pPB-t-rtTA. (**F**) A HeLa cell line stably expressing rtTA was transfected with pHIV.Gag-SNAP-tag rtTA and incubated with SNAP-tag ligand JF549.

Previous studies have shown that HIV-1 Gag is present in the nucleus [18,19]. To determine whether HIV-1 Gag trafficked to the nucleus regardless of cell type or fusion tag, we examined HeLa HIV.Gag-GFP rtTA cells [19,26], a HeLa cell line expressing a stably integrated, dox-inducible HIV-1 provirus encoding a Gag-GFP fusion protein (Figure 1A). Following induction, Gag foci were observed in nucleus, cytoplasm and at the plasma membrane (Figure 1A,C). To determine whether the same Gag localization was seen with transient transfection, HeLa, U2OS, or Hela rtTA cells were transfected with dox-inducible proviral constructs expressing either Gag-CFP or Gag-SNAP-tag fusion (pHIV.Gag-CFP rtTA and pHIV.Gag-SNAP-tag rtTA, respectively; Figure 1B,D–F). pHIV.Gag-CFP rtTA was modified from a plasmid kindly provided by Nathaniel Sherer [33]. This plasmid was transiently co-transfected with pPB-t-rtTA into HeLa and U2OS cells, and Gag-CFP foci were observed in nucleus, cytoplasm, and plasma membrane (Figure 1D,E). To ensure that HIV-1 Gag nuclear localization was not a phenomenon related to the GFP or CFP tags, we fused the SNAP-tag to the C terminus of Gag, which provides single molecule resolution of proteins when cells are incubated with JF549 SNAP-tag ligand [39]. HeLa rtTA cells transfected with pHIV.Gag-SNAP-tag rtTA JF549 demonstrated a very similar distribution of Gag foci in the nucleus, cytoplasm, and plasma membrane (Figure 1F) when compared to the HeLa HIV.Gag-GFP rtTA cell line.

### 3.2. HIV-1 Gag Is Present within the Nuclear Fractions Using Biochemical Methods

**Figure 2:**
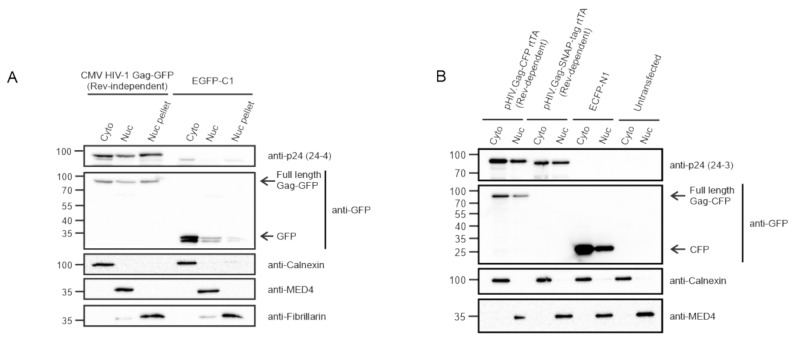
Presence of HIV-1 Gag in cytoplasmic and nuclear subcellular fractions. (**A**) Hela cells transfected with CMV HIV-1 Gag-GFP and EGFP-C1 plasmids were fractionated into cytoplasmic (Cyto), nucleoplasm (Nuc), and nuclear pellet (Nuc pellet) and analyzed by immunoblot using anti-p24 and anti-GFP antibodies. EGFP-C1 serves as negative control. Fraction purity was assessed using Calnexin (cytoplasm), MED4 (nucleoplasm), and fibrillarin (nucleolar, nuclear pellet). (**B**) Immunoblot of HeLa rtTA cell cytoplasmic and nuclear fractions following transfection with either pHIV.Gag-CFP rtTA, pHIV.Gag-SNAP-tag rtTA, or ECFP-N1 compared to untransfected cells. Fractions were probed using anti-p24 and anti-GFP antibodies for the presence of HIV-1 Gag. ECFP-N1 transfection and untransfected samples serves a negative control. Purity of the fractions was assessed using Calnexin (cytoplasmic) and MED4 (nuclear) antibodies.

To determine whether HIV-1 Gag would be detected in the nucleus using a biochemical approach, we initially used a Rev-independent HIV-1 Gag-GFP (CMV HIV-1 Gag-GFP) construct, which was transfected into HeLa cells and fractionated into cytoplasmic and nuclear compartments. Immunoblotting with anti-p24 and anti-GFP antibodies detected the presence of full-length Gag-GFP in both fractions, as well as the insoluble nuclear pellet (Figure 2A). Controls for each fraction were performed using cellular antibodies for Calnexin (cytoplasm), MED4 (nucleus), and fibrillarin (nuclear pellet). Next, HeLa rtTA cells transfected with either the Rev-dependent pHIV.Gag-CFP rtTA or pHIV.Gag-SNAP-tag rtTA constructs were subjected to subcellular fractionation and immunoblotting with anti-p24 and ant-GFP antibodies, demonstrating that HIV-1 Gag was present in cytoplasmic and nuclear fractions (Figure 2B). Fraction purity was assessed using cellular antibodies for Calnexin (cytoplasm) and MED4 (nucleus). Moreover, cells were transfected with either EGFP-C1 (Figure 2A) or ECFP-N1 (Figure 2B) as a control for the presence of free fluorophore in transfected cells. The absence of free GFP and CFP in the Gag fractions demonstrated that full-length Gag was present in the nucleus and cytoplasm.

### 3.3. HIV-1 Gag Specifically co-Localizes with USvRNA in Discrete Nuclear Foci

**Figure 3:**
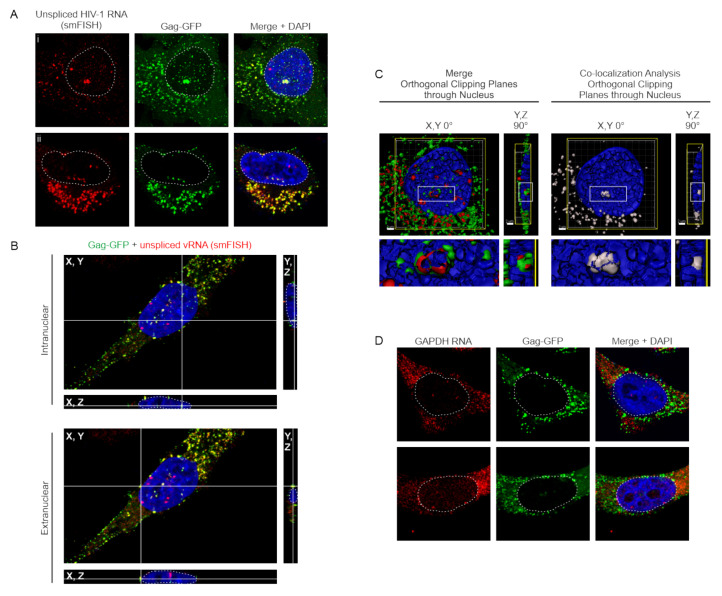
HIV-1 Gag co-localizes with USvRNA in the nuclei of HeLa HIV.Gag-GFP rtTA cells. (**A**) Two different cells (panels i and ii) showing co-localization of HIV-1 Gag-GFP (green), USvRNA (red, detected by smFISH), and co-localized foci (yellow) in the nucleus (DAPI-stained, shown as blue, outlined in white), cytoplasm, and along the plasma membrane of dox-induced HIV-1 Gag-GFP rtTA cells. (**B**) Cross-sections of dox-induced HeLa HIV.Gag-GFP rtTA cells containing nuclear HIV-1 Gag-GFP foci co-localized with USvRNA within intranuclear and extranuclear space, as indicated. Gag-USVRNA co-localized foci located at the intersection of the white cross hairs) are depicted in X, Y (center); Y, Z (right); and X, Z (bottom) planes, with the DAPI-stained nucleus (blue) outlined with a white dashed line. (**C**) Left, Three-dimensional surface rendering exhibiting HIV-1 Gag (green) and USvRNA (red) co-localization within a single orthogonal clipping through the XY plane of the nucleus. Right, Co-localization analysis of Gag-USvRNA co-localized foci (white) using Imaris. Images were depicted in the XY (center) and YZ (rotated 90°) planes with scale bars = 2 µm (white). vRNP foci can be seen located in the perichromatin (DAPI-poor) space. (**D**) GAPDH RNA (detected using smFISH; red) and HIV-1 Gag-GFP (green) have negligible co-localization within the nucleus (DAPI-blue, white outline).

Our RSV data shows that RSV Gag co-localizes with USvRNA in the nucleus [16], so we asked whether HIV-1 nuclear Gag also co-localized with USvRNA. Visualization of USvRNA in the HeLa HIV.Gag-GFP rtTA cell line was achieved using single-molecule fluorescence in situ hybridization (smFISH) probes tiled against the *gag* coding region, thus specifically detecting unspliced transcripts. Confocal imaging of single Z-slices through the widest nuclear diameter of cells containing Gag-GFP (green) and USvRNA (red) signal permitted the detection of florescent foci within the nucleus. Two-dimensional quantitative imaging analysis using custom MATLAB scripts demonstrated that 65% of all HeLa HIV.Gag-GFP rtTA cells that expressed Gag, (N = 121) imaged across five replicate experiments, contained nuclear HIV-1 Gag-GFP. In the cells that had nuclear HIV-1 Gag, an average of 5.6 ± 0.6 Gag-GFP and 9.1 ± 0.8 USvRNA nuclear foci were present. Two-dimensional co-localization analysis revealed that 39.0 ± 3.7% of the nuclear HIV-1 Gag-GFP foci co-localized with USvRNA foci and 19.6 ± 2.2% of USvRNA foci co-localized with the nuclear HIV-1 Gag-GFP foci (Figure 3A; two different cells shown in panels i and ii). A three-dimensional reconstruction of the z-stack of the cell in Figure 3C panel i was shown in Video S1, in which scrolling through each individual z-plane showed the positions of vRNPs throughout the cell, with the nucleus appearing as blue (DAPI-stained). To highlight vRNPs (co-localized Gag-USvRNA foci), a co-localization channel was generated, and Video S2 shows vRNPs (white) present in each plane of the same cell.

To validate that Gag-USvRNA co-localization (yellow) was occurring within the nucleus in three-dimensions, confocal Z-stacks were reconstructed and cross-sections were created showing co-localized foci of interest in the X, Y; Y, Z; and X, Z planes located within the DAPI signal of the nucleus (white outline) (Figure 3B). vRNPs (yellow foci) were present in both intranuclear and extranuclear locations, and is readily differentiated between these two compartments. In addition, the Z-stack from the cell presented in Figure 3A (panel i) was used to generate a three-dimensional surface rendering, which was subjected to orthogonal clipping planes to bisect the nuclei in the X, Y or Y, Z plane to reveal HIV-1 Gag-GFP (green) and USvRNA (red) co-localized foci within the DAPI-poor perichromatin space (Figure 3C, left and Video S3) in the nucleus (blue). A white co-localization channel was generated to visualize only co-localized Gag-USvRNA complexes (Figure 3C, right). The enlarged image below each panel shows more clearly that the vRNPs are in the perichromatin space.

To further examine whether HIV-1 Gag would co-localize non-specifically with a cellular mRNA, we conducted similar studies using smFISH probes targeted against the human GAPDH transcript. Two-dimensional co-localization analysis of cells (N = 40) from three replicate experiments revealed that on average cells contained 7.3 ± 0.9 HIV-1 Gag-GFP foci and 2.0 ± 0.1 GAPDH RNA foci per nucleus (Figure 3D). These cells showed a negligible degree of co-localization (~0.4 ± 0.3% Gag with RNA) between nuclear HIV-1 Gag-GFP and GAPDH RNA foci (Figure 3D). Almost all of the cells (38/40) lacked co-localized Gag and vRNA foci. The other two cells had a single focus of GAPDH RNA that co-localized with Gag; a Grubbs’ test deemed these two foci as outliers. These data suggest that HIV-1 Gag co-localization with USvRNA in the nucleus is sequence specific.

### 3.4. HIV-1 Gag Associates with USvRNA in the Nucleus

**Figure 4:**
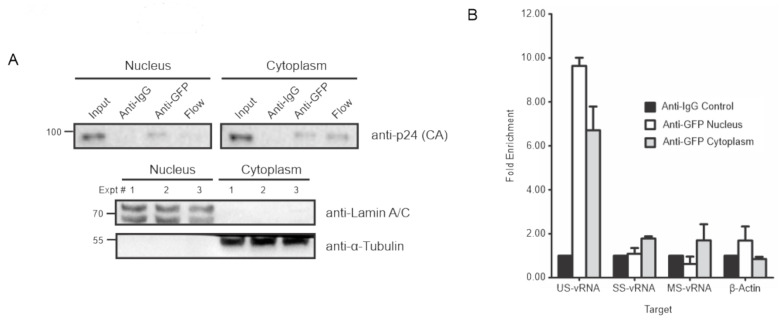
HIV-1 Gag specifically associates with USvRNA in nuclear and cytoplasmic fractions. (**A**) HeLa HIV.Gag-GFP rtTA cell fractions were immunoprecipitated using anti-GFP antibodies and detected for the presence of HIV-1 Gag via p24. Anti-IgG immunoprecipitation serves as negative control. Fraction purity was assessed using Lamin A/C and α-tubulin. (**B**) Immunoblot analysis of HIV-1 Gag-GFP immunoprecipitated from cytoplasmic and nuclear fractions of induced HeLa HIV.Gag-GFP rtTA cells. RT-qPCR analysis of targeted unspliced vRNAs (US), singly-spliced vRNAs (SS), multiply-spliced vRNAs (MS), and beta-actin RNAs with HIV-1 Gag-GFP immunoprecipitated from fractionated HIV-1 Gag-GFP rtTA cells.

Although the co-localization data suggested that Gag associates with USvRNA in the nucleus, to examine the association using an independent assay, RNA-immunoprecipitation was performed on fractionated HeLa HIV.Gag-GFP rtTA cells. Dox-induced HeLa HIV.Gag-GFP rtTA cells were fractionated into nuclear and cytoplasmic compartments, as confirmed by anti-Lamin A/C and anti-α-tubulin immunoblots, respectively, to test for purity (Figure 4A). HIV-1 Gag-GFP was immunoprecipitated from each compartment using an anti-GFP antibody. Immunoblot analysis of immunoprecipitated proteins with anti-p24 Gag hybridoma demonstrated Gag-GFP pulled down from the nuclear and cytoplasmic fractions with no pulldown using an IgG control for specificity (Figure 4A). Purified RNA extracted from each immunoprecipitation was used to generate cDNA for real-time quantitative PCR of vRNA [US, singly-spliced (SS), multiply-spliced (MS)] and β-actin housekeeping control mRNA targets [26]. USvRNA was enriched nearly ten-fold in the nuclear compartment and approximately seven-fold in the cytoplasmic compartment, indicating a specific association of HIV-1 Gag-GFP with USvRNA in both compartments. By contrast, SSvRNA, MSvRNA, and β-actin mRNA yielded no enrichment over the non-specific control IgG (Figure 4B). Additionally, GFP immunoprecipitations performed in TNFα treated HIV-R7/E-/GFP Jurkat cells, containing an integrated modified HIV-1 provirus expressing GFP in place of the Nef reading frame [37], yielded no enrichment of vRNA or β-actin mRNA targets over background in either compartment (data not shown). Together these data suggest the association of HIV-1 Gag-GFP with USvRNA is specific and not a result of GFP binding to vRNA non-specifically.

### 3.5. Effects of Transcription Inhibition on HIV-1 Gag/USvRNA Co-Localization

Based on previous studies demonstrating that HIV-1 Rev associates with USvRNA co-transcriptionally [48,49], we examined whether inhibiting transcription would alter HIV-1 Gag-GFP-USvRNA nuclear co-localization. To explore this possibility, dox-induced HeLa HIV.Gag-GFP rtTA cells were treated with either of the transcription inhibitors DRB or Act D for 30 or 60 min. Upon completion of quantitative two-dimensional imaging analysis, we found in three replicate experiments that 95.7% of cells (N = 47) treated with DRB for 30 min contained nuclear HIV-1 Gag-GFP foci. These cells showed a significant increase in the average number of nuclear HIV-1 Gag-GFP foci (10.9 ± 1.5) and no change in the number of nuclear USvRNA foci (9.3 ± 1.1) compared to the untreated control (5.6 ± 0.6 HIV-1 Gag-GFP and 9.1 ± 0.8 USvRNA nuclear foci; Figure S1A,B). Conversely, the 60 min treatment with DRB resulted in a significant decrease in both nuclear HIV-1 Gag-GFP (3.5 ± 0.4) and USvRNA (6.1 ± 1.0) foci compared to the untreated control (Figure S1A,B). These data suggest that initially, there is an increase in the percent of cells with nuclear Gag and the number of Gag foci per nucleus, yet over time, transcription inhibition due to DRB treatment resulted in a decrease in the number of HIV-1 Gag-GFP and USvRNA nuclear foci.

Additionally, nuclear HIV-1 Gag-GFP foci were found in 59.0% of cells (N = 39) treated for 30 min with Act D. These cells did not exhibit a significant change in the average number of nuclear HIV-1 Gag-GFP foci (3.9 ± 1.0), but did show significant decrease in the number of USvRNA nuclear foci (4.0 ± 0.8) compared to the control cells (Figure S1A,C). Likewise, 54.8% of cells (N = 42) treated for 60 min with Act D contained nuclear HIV-1 Gag-GFP foci and displayed a significant decrease in the number of HIV-1 Gag-GFP (2.9 ± 0.4) and USvRNA (5.8 ± 0.9) nuclear foci compared to the control cells (Figure S1A,C). Similar to DRB treatment, this data also suggests that over time transcription inhibition with Act D decreased the number of HIV-1 Gag-GFP and USvRNA nuclear foci.

Together these findings demonstrate that treatment with either transcription inhibitor results in a decrease in the number of nuclear HIV-1 Gag-GFP and USvRNA foci over time, compared to the untreated control (Figure S1). This decrease in the number of nuclear foci may be due to either fewer HIV-1 Gag-GFP and USvRNA molecules being present in the nucleus following transcription inhibition or the accumulation of these nuclear molecules into fewer discrete regions resulting in a reduced number of individual foci. Although the general trend of transcription inhibition resulted in decreased numbers of nuclear HIV-1 Gag-GFP and USvRNA foci, an increase was seen in the percent of cells containing nuclear HIV-1 Gag-GFP foci and the number of those foci during the 30 min DRB treatment, which suggests an initial accumulation of total nuclear HIV-1 Gag-GFP prior to its concentration into a limited number of foci.

**Figure 5:**
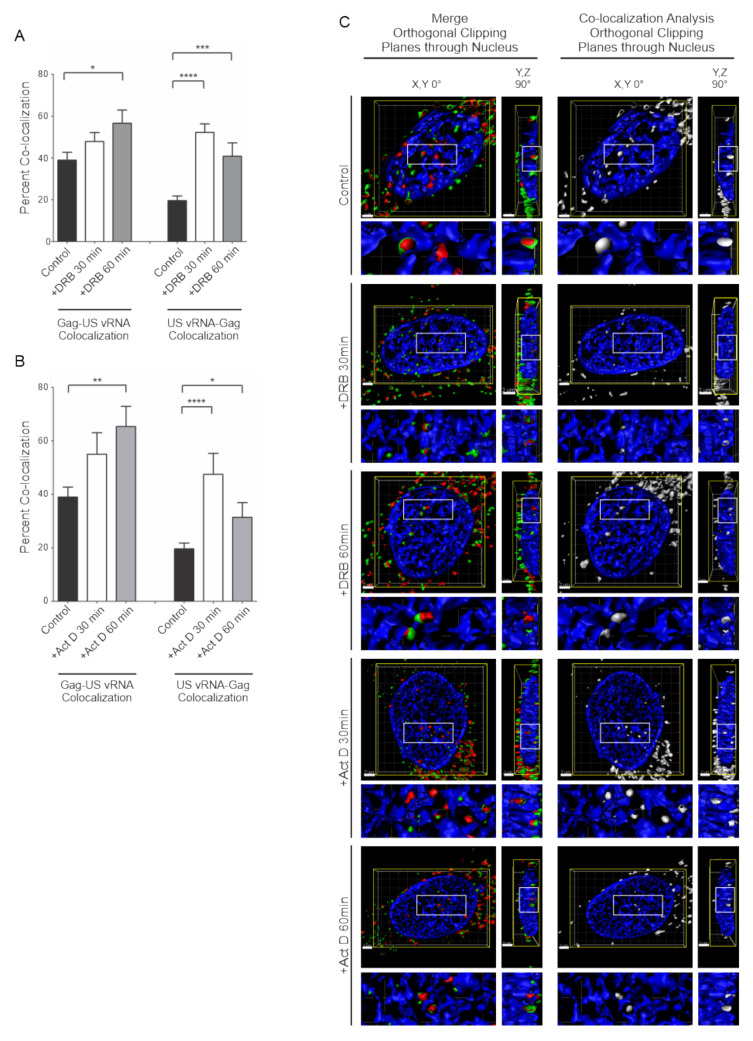
Transcription inhibition of HeLa HIV.Gag-GFP rtTA cells decreased the number of nuclear HIV-1 Gag and USvRNA foci but increased the degree of co-localization. Quantitative analysis of dox-induced HeLa HIV.Gag-GFP rtTA cells demonstrating the mean percent of nuclear HIV-1 Gag co-localization with USvRNA and USvRNA co-localization with HIV-1 Gag following transcription inhibition with DRB (**A**) or Act D (**B**) for 30 and 60 min compared to the untreated control. (error bars = standard error of the mean, statistical significances: * *P* < 0.05, ** *P* < 0.01, *** *P* < 0.001, **** *P* < 0.0001). (**C**) 3D surface renderings of dox-induced HeLa HIV.Gag-GFP rtTA cells depicting the co-localization (shown in white) of HIV-1 Gag-GFP (green) and USvRNA (red, detected by smFISH) foci within the nucleus (DAPI, blue) following treatment with the transcription inhibitor DRB or Act D for 30 and 60 min. 3D surface renderings were subjected to an orthogonal clipping plane to bisect the nucleus in XY or YZ planes. The white rectangle represents the region of interest enlarged below.

To examine whether HIV-1 Gag-GFP and USvRNA co-localization was affected by interfering with transcription dynamics, cells treated with DRB or Act D were assessed for quantitative two-dimensional co-localization, as previously described. Cells treated with DRB for 30 min exhibited minimal change in the percent of nuclear HIV-1 Gag-GFP foci co-localized with USvRNA (47.8 ± 4.3%) but displayed a significant increase in USvRNA molecules that were co-localized with nuclear HIV-1 Gag-GFP (52.2 ± 4.0%) compared to the control (39.0 ± 3.7%, HIV-1 Gag-GFP/USvRNA; and 19.6 ± 2.2%, USvRNA/HIV-1 Gag-GFP nuclear co-localization) (Figure 5A). Following 60 min DRB treatment, a significant increase could be seen in both the percent of HIV-1 Gag-GFP/USvRNA co-localization (56.6 ± 6.3%) and in USvRNA/HIV-1 Gag-GFP nuclear co-localization (40.8 ± 6.4%) compared to the control (Figure 5A). Similarly, treatment of cells with Act D for 30 min resulted in minimal change in the percent of HIV-1 Gag-GFP/USvRNA co-localization (55.0 ± 8.1%) throughout the cell yet a significant increase in USvRNA/HIV-1 Gag-GFP nuclear co-localization (47.5 ± 7.9%) (Figure 5B). Act D treatment for 60 min produced a significant increase in both the percent of HIV-1 Gag-GFP/USvRNA co-localization (65.3 ± 7.6%) and in USvRNA/HIV-1 Gag-GFP nuclear co-localization (31.4 ± 5.5%) compared to the control cells (Figure 5B).

In addition to the two-dimensional co-localization analysis, HIV-1 Gag-GFP and USvRNA co-localization during control and treatment conditions were also confirmed to exist in three-dimensions through the use of surface rendering reconstructions generated from confocal Z-stacks (Figure 5C and Figure S1A). Orthogonal clipping planes were made to show the Y, Z dimension with green and red foci in the left panels, with co-localized foci indicating Gag-USvRNA complex formation. To better visualize Gag-USvRNA complexes, co-localization analysis was performed in three-dimensions using the surface renderings, demonstrating vRNP foci (white; right panels). The enlarged panels below each image demonstrate that the vRNPs were located in the perichromatin space in all cases. Given the increase in co-localization observed following treatment with either transcription inhibitor, these results strongly suggest that HIV-1 Gag-GFP associates with USvRNA in a transcription-dependent fashion, possibly through a co-transcriptional mechanism.

### 3.6. Effects of LMB Treatment on HIV-1 Gag/USvRNA Association

**Figure 6:**
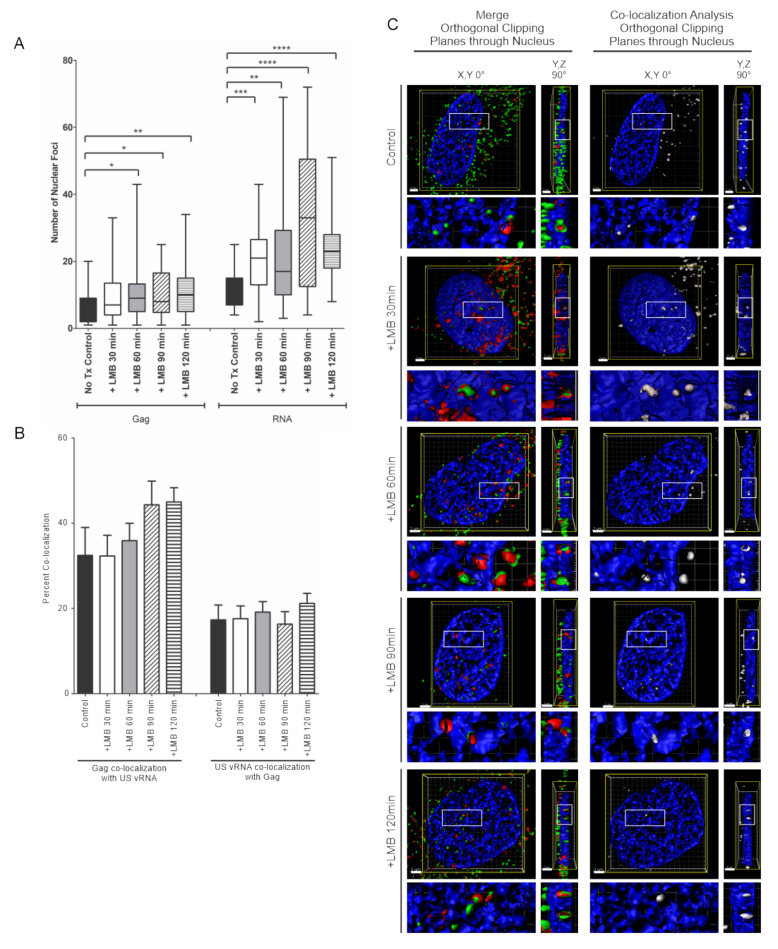
Inhibiting CRM1-mediated nuclear export in HeLa HIV.Gag-GFP rtTA cells increased HIV-1 Gag and USvRNA nuclear foci but did not affect the degree of co-localization between HIV-1 Gag and USvRNA. (**A**) Quantitative analysis depicting the mean number of HIV-1 Gag-GFP and USvRNA nuclear foci in dox-induced HeLa HIV.Gag-GFP rtTA cells following LMB treatment compared to the untreated control cells. (**B**) Quantitative analysis of dox-induced HIV-1 Gag-GFP rtTA cells demonstrating the mean percent of nuclear HIV-1 Gag co-localization with USvRNA and USvRNA co-localization with HIV-1 Gag following treatment with LMB for 30, 60, 90, or 120 min prior to fixation compared to the untreated control. (error bars = standard error of the mean, statistical significances: * *P* < 0.05, ** *P* < 0.01, *** *P* < 0.001, **** *P* < 0.0001). (**C**) 3D surface renderings of dox-induced HeLa HIV.Gag-GFP rtTA cells depicting the co-localization (shown in white) of HIV-1 Gag-GFP (green) and USvRNA (red, detected by FISH) foci within the nucleus (DAPI, blue) following treatment with LMB. 3D surface renderings were subjected to an orthogonal clipping plane bisected in the X, Y and Y, Z planes to show the co-localization of foci within the nucleus. The white rectangle represents the region of interest enlarged in the panels below.

Inhibiting transcription decreased the number of nuclear HIV-1 Gag-GFP and USvRNA foci but resulted in an increase in co-localization, suggesting that the association of HIV-1 Gag and USvRNA is augmented by halting transcription. Following transcription, HIV-1 USvRNA is exported from the nucleus by the Rev protein in a CRM1-dependent fashion. Therefore, we asked whether interfering with the Rev-CRM1 association would result in the nuclear accumulation of HIV-1 Gag-USvRNA complexes. To address this question, dox-induced HeLa HIV.Gag-GFP rtTA cells were treated with the CRM1 inhibitor leptomycin B (LMB) for 30, 60, 90, or 120 min prior to fixation. Quantitative assessment demonstrated a marked increase in the number of nuclear USvRNA foci compared to untreated control cells (11.8 ± 1.2, control; 20.9 ± 1.8, 30 min, *P* < 0.001; 22.6 ± 2.9, 60 min, *P* < 0.01; 33.7 ± 3.9, 90 min, *P* < 0.0001; 24.4 ± 1.7, 120 min, *P* < 0.0001) (Figure 6A). In addition, a significant increase was also observed in the average number of HIV-1 Gag-GFP nuclear foci with LMB treatment compared to control cells for each timepoint except for 30 min (6.2 ± 1.1 control; 10.0 ± 1.4, 30 min; 10.9 ± 1.5, 60 min, *P* < 0.05; 10.2 ± 1.3, 90 min, *P* < 0.05; 11.2 ± 1.2, 120 min, *P* < 0.01) (Figure 6A). This result suggests that HIV-1 Gag nuclear export may be linked to the export of Rev or a cellular CRM1-dependent factor because HIV-1 Gag itself does not appear to contain an intrinsic NES that is sensitive to CRM1 inhibition [18,21].

Although there was an increase in the number of HIV-1 Gag-GFP and USvRNA nuclear foci with LMB treatment, there was no significant change in either the percent of nuclear HIV-1 Gag-GFP co-localization with USvRNA (32.5 ± 6.6%, control; 32.3 ± 4.9%, 30 min; 35.9 ± 4.1%, 60 min; 44.3 ± 5.5%, 90 min; 45.0 ± 3.4%, 120 min) or USvRNA co-localization with nuclear HIV-1 Gag-GFP (17.3 ± 3.5%, control; 17.6 ± 3.0%, 30 min; 19.1 ± 2.5%, 60 min; 16.3 ± 2.9%, 90 min; 21.2 ± 2.4%, 120 min) compared to untreated control cells (Figure 6B). Nuclear co-localization between HIV-1 Gag-GFP and USvRNA during LMB treatment was also shown to exist in three-dimensions as illustrated by surface renderings (Figure 6C and Figure S2) undergoing orthogonal clipping to show the *en face* and 90° rotations in merged images (red/green foci) and after applying the co-localization algorithm (white foci), as described in Figure 5. In combination, the data using transcription inhibitors and LMB treatment suggest that the association of HIV-1 Gag with USvRNA occurs prior to the vRNP becoming competent for nuclear export.

### 3.7. Nuclear and Chromatin Associated Localization of HIV-1 Gag and Rev

**Figure 7:**
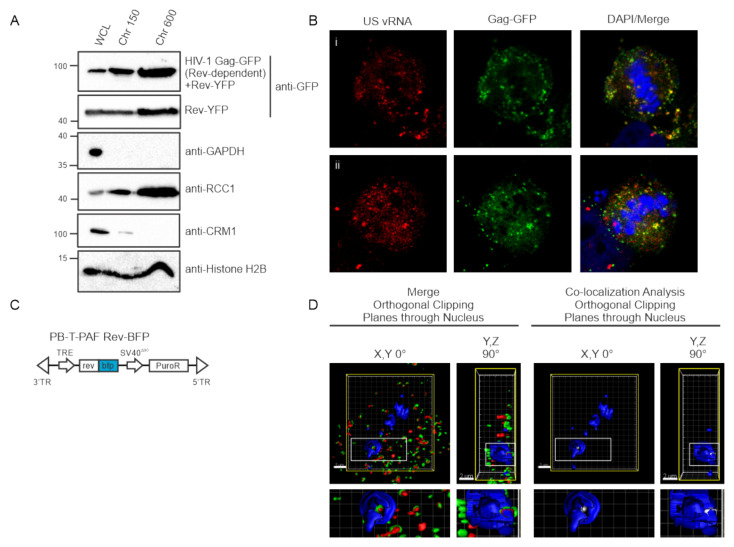
HIV-1 Gag and Rev are both present in chromatin-associated fractions and are co-localized in three dimensions. (**A**) 293T cells transfected with HIV-1 Gag-GFP with or without Rev-YFP expression plasmids were separated into chromatin-associated protein fractions representing euchromatin (Chr 150) and heterochromatin (Chr 600) and analyzed by immunoblotting using anti-GFP antibodies. Fraction purity was assessed by immunoblotting with antibodies to detect GAPDH (present only in whole cell lysate, WCL), CRM1 (WCL and chromatin), RCC1 (WCL and chromatin) and Histone H2B (WCL and chromatin). (**B**) Localization of HIV-1 Gag-GFP (green) and USvRNA (red, detected by smFISH) in two dividing cells (i and ii) showing persistence of Gag-USvRNA co-localization during metaphase but no binding to the chromosome, which was stained blue using DAPI. (**C**) Schematic diagram of dox-inducible Rev-BFP used to create HeLa HIV.Gag-GFP rtTA Rev-BFP cells. (**D**) 3D-surface renderings of induced HeLa HIV.Gag-GFP rtTA Rev-BFP cells depicting three-way co-localization in merged images of Gag-GFP (green) and USvRNA (red, detected by smFISH) within the Rev-BFP (blue) mask using orthogonal clipping planes through the nucleus of 0° and 90° rotation. The large white square is the frame that denotes the cell as three-dimensional, the yellow square indicates that the cell was subjected to the orthogonal clipping plane, and the small white rectangle represents the region of interest that was enlarged in the panel below. A co-localization algorithm was used to demonstrate Gag and USvRNA co-localization (white foci) within the confines of the Rev (blue) signal. The small rectangle was enlarged below to provide additional detail.

Based on evidence presented herein that HIV-1 Gag-GFP co-localizes with USvRNA during or just after transcription (Figure 3, Figure 5 and Figure 6), we wished to determine whether Gag was present in the chromatin-associated protein fraction (perichromatin), which contains transcription machinery [50,51]. To investigate this possibility, we performed biochemical fractionations and extracted proteins from isolated chromatin fractions using two different conditions in 293T cells. Extraction of the chromatin fractions using low salt (150 mM NaCl, Chr 150) elutes factors involved in transcriptionally active euchromatin (Chr 150) whereas high salt (600 mM NaCl, Chr 600) extracts proteins bound to transcriptionally inactive heterochromatin. We found that HIV-1 Gag was present in both euchromatin and heterochromatin associated protein fractions (Figure 7A). In addition, we detected Rev, which interacts with USvRNA during transcription [19,48] in both fractions at a similar ratio as Gag (Figure 7A). To examine whether HIV-1 Gag was directly bound to chromatin, as reported for PFV Gag [25,52,53] and the MLV Gagp12 protein [54,55,56], we examined the localization of Gag and USvRNA in HeLa HIV.Gag-GFP rtTA cells undergoing cell division. Interestingly, although both USvRNA and Gag remained localized in discrete foci and continued to be co-localized in metaphase cells, the vRNPs remained in the perichromatin space and throughout the cell, but did appeared to bind directly to the chromosome as seen for PFV (Figure 7B).

### 3.8. Three-Dimensional Co-Localization of HIV-1 Gag, USvRNA, and Rev

Given that HIV-1 Rev binds USvRNA co-transcriptionally [43,57] and was shown to be associated with Gag in nucleoli [19], we asked whether there is an interaction of HIV-1 Gag, USvRNA, and Rev that could be visualized *in situ*. To test this possibility, we modified the HeLa HIV.Gag-GFP rtTA cell line by using *piggyBac* transposon-mediated gene transfer to integrate a dox-inducible Rev-BFP into the cellular chromosome (Figure 7C). Following induction and fixation of HeLa HIV.Gag-GFP rtTA Rev-BFP cells, a confocal Z-series of cells containing nuclear HIV-1 Gag-GFP foci were acquired and a 3D surface rendering was generated using Imaris imaging analysis software. A region of interest was created by masking the Rev-BFP signal, and a co-localization channel within the Rev-BFP mask was produced for HIV-1 Gag-GFP and USvRNA, as described [44] (Figure 7D). Three-dimensional co-localization between HIV-1 Gag-GFP, USvRNA, and Rev-BFP was visualized in 43% of cells imaged (N = 28, Figure 7D), demonstrating that all three molecules localized together in this region of the nucleus, indicating that the association between HIV-1 Gag and Rev is likely mediated by USvRNA.

### 3.9. Co-Localization of HIV-1 Gag and USvRNA in JLat 10.6 CD4+ T Cells

**Figure 8:**
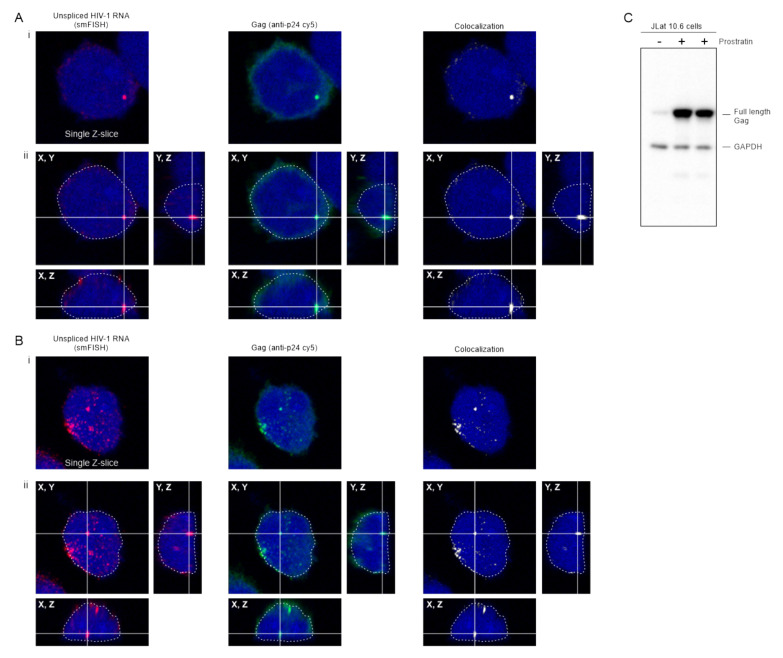
HIV-1 Gag and co-localizes with USvRNA in the nucleus of JLat 10.6 cells. (**A**,**B**) Confocal images showing (**i**) single z-slices and (**ii**) cross-sections of three-dimensional reconstructions of two examples of JLat 10.6 cells activated by prostratin. USvRNA labeled via smFISH (red) is co-localized with full-length HIV-1 Gag (anti-p24 cy5 labeled; green) in the nucleus outlined in white dashed line (DAPI, blue). A co-localization channel (white) was generated to show that the Gag and USvRNA signals are present in the same pixels of the image. In each cell, a vRNP consisting of USvRNA and Gag was present within a crosshair to show its location within the nucleus in three planes. (**C**) Immunoblot of JLat 10.6 whole cell lysates in the absence (-) or presence (+) of prostratin. The full-length Gag polyprotein was present in cells reactivated from latency. GAPDH served as a loading control.

The HIV constructs in the experiments up to this point were expressed using doxycycline-inducible promoters, therefore we examined whether the results could be confirmed in a more biologically relevant cell type in which the native viral 5′UTR was intact. To this end, we used JLat 10.6 cells, which are Jurkat-derived T cells infected with a full-length HIV-1 genome that contains *gfp* in place of *nef* and a frameshift in the *env* gene [37]. JLat 10.6 cells were previously thought to contain a single integration site [58], although more recently were shown to have two proviral integration sites, one of which may be incomplete or defective [59]. The HIV-1 provirus is latent in these cells and can be reactivated using a variety of drugs [37]. In Figure 8, JLat 10.6 cells were treated with prostratin, which induces expression through the NF-κB pathway [60,61,62]. Confocal z-stacks were obtained in which HIV-1 USvRNA was labeled using smFISH and Gag was detected by immunofluorescence. HIV-1 USvRNA (red) co-localized with Gag (green) in the nucleus (DAPI; outlined in white) in a large focus that likely represents a burst of transcription occurring at the proviral integration site (Figure 8A, panel i), as shown previously [59]. The lower panel (Figure 8B, panel i) depicts a different cell showing Gag co-localization with USvRNA at two large transcription sites, as well as several additional smaller vRNP complexes throughout the nucleoplasm with accumulation near the nuclear periphery, similar to USvRNA imaged in HIV-1 infected cells [63]. To demonstrate that these foci were contained within the nucleus, a three-dimensional reconstruction (Figure 8A,B, panel ii) was performed, showing that the complexes were located within the DAPI signal in all three aspects. A co-localization algorithm was used to identify Gag-USvRNA complexes (white), highlighting larger vRNPs as well as the smaller co-localized complexes within the nucleus.

Because *env* is defective in JLat 10.6 cells, there is no reinfection of the cells, so mature CA is not expected to be present. To examine whether the Gag protein expressed in these cells was full-length, activated JLat 10.6 cells were lysed and subjected to Western blot analysis (Figure 8C). Full-length Gag was observed in the cell lysates, indicating that the imaging studies detected full-length Gag and not free CA. Additionally, due to inability of released virus particles to reinfect the cells, the labeled vRNA is not from incoming virus particles, but rather is derived from newly synthesized USvRNA in cells reactivated from latency. Although the integrated provirus is not being labeled in these experiments, previous work has identified that the large vRNA foci in these cells is the transcriptional burst of vRNA synthesis that occurs at the proviral integration site [63]. Together, these data suggest that wild-type HIV-1 Gag co-localization with USvRNA occurs in the setting of latency reversal in CD4+ T cells, independent of doxycycline-induction of expression or fusion of Gag to a fluorescent tag.

## 4. Discussion

An outstanding question in the field of retrovirology is how and where the Gag protein selects USvRNA as a genome for packaging into virions. We recently presented evidence that RSV Gag associates with USvRNA at viral transcription sites to form a vRNP that traverses the nuclear envelope, suggesting that this nuclear vRNP may play a role in genomic RNA packaging [9,11,12,13,14,15,16,17]. To follow up earlier reports that HIV-1 Gag localizes to the nucleus [18,19] and to determine whether HIV-1 also forms vRNPs in the nucleus, we studied a dox-inducible HIV-1 provirus encoding Gag fused to GFP, CFP, or SNAP-tag, and used smFISH for detection of USvRNA. The results demonstrated that Gag associates with USvRNA in discrete foci in the nucleus to form vRNPs in HeLa cells. Furthermore, in CD4+ T cells (JLat 10.6) reactivated from latency, HIV-1 Gag co-localized with USvRNA at the transcriptional burst site, demonstrating that Gag binds newly transcribed vRNA to form vRNP complexes. Others have shown that HIV-1 Gag associates with vRNA in the nucleus [8,59], although no specific comments were made discussing the meaning of these previous observations, up to now.

**Figure 9:**
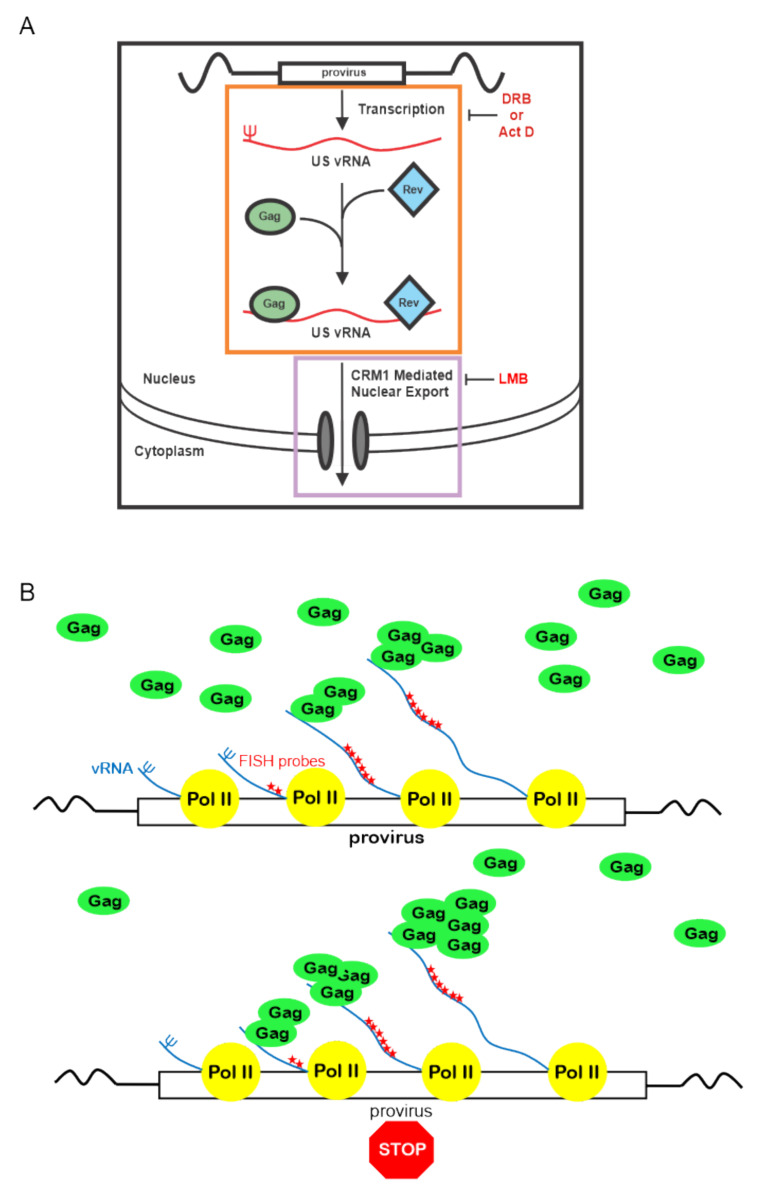
Proposed model of HIV-1 Gag association with USvRNA during transcription. (**A**) Our results demonstrate that treatment of HeLa HIV.Gag-GFP rtTA cells with Act D or DRB caused Gag to accumulate on USvRNAs that may be at or near transcription sites (orange box). Because Rev also binds USvRNA co-transcriptionally, there is a possibility that both Gag and Rev associate with USvRNA at transcription sites. Nuclear export of USvRNA was inhibited by LMB (purple box), along with small increase in HIV-1 Gag-GFP nuclear foci, yet there was no change in the degree of Gag-USvRNA co-localization. This finding suggests that there is a narrow “window of opportunity” for Gag-USvRNA to form vRNP complexes, starting at transcription but ending prior to formation of the export complex. (**B**) The model proposes that Gag binds to the 5′UTR psi sequence as it emerges during transcription. With transcription inhibition, Gag accumulates on stalled transcription complexes leading to an increase in Gag-USvRNA co-localization, potentially through additional Gag-Gag or Gag-RNA interactions.

In the current report, we have dissected the Gag-USvRNA nuclear interaction in more detail, demonstrating that HIV-1 vRNP formation is enhanced with transcriptional inhibition, suggesting that Gag accumulates at stalled viral transcription sites. Based on these results, we propose a novel model for the initial interaction of HIV-1 Gag with genomic RNA, as depicted in Figure 9. As nascent viral RNAs are transcribed at the proviral integration site, the 5′ end containing the psi packaging sequence would emerge first, and once it adopts the proper three-dimensional conformation, it would be available for Gag binding. Shutting off transcription during elongation with Act D or DRB would lead to an accumulation of vRNA with intact 5′ ends, allowing Gag to bind to psi, manifesting as an increase in co-localization in our imaging studies. This finding suggests that nascent HIV-1 RNPs are stabilized under these conditions. In contrast to this result, interfering with transcriptional elongation reduces Rev binding to viral RNA [49,64] possibly because transcription is halted prior to synthesis of the RRE at the 3′ end of the genome. Future studies comparing these observations with deletions in psi and mutations in Gag that affect vRNA binding will be needed to further examine the *cis* and *trans*-acting elements required for the interaction of Gag with USvRNA in the nucleus.

Our study also revealed that HIV-1 Gag was present in euchromatin fractions using biochemical methods (Figure 7A), and 3D imaging data demonstrated that Gag-USvRNP complexes are nestled in the perichromatin region, just outside of the DAPI-stained condensed chromatin (see Figure 3C, Figure 5C and Figure 6C). The perichromatin region contains transcriptionally active genes and an abundance of transcription, splicing, RNA binding proteins, and chromatin modifying factors [50,51]. Several proteomic experiments have identified chromatin modifiers, splicing factors, and transcriptional regulators that interact with HIV-1 Gag [65,66,67,68,69]. It will be informative to determine whether any of these previously identified chromatin-associated binding partners of Gag play a role in vRNP formation, trafficking, export, or packaging.

The prior studies indicating that HIV-1 Rev associates with USvRNA co-transcriptionally [48,49] led us to evaluate whether Gag, Rev, and USvRNA were present together in the nucleus. We found that Gag and Rev were isolated biochemically in the euchromatin and heterochromatin fractions in similar ratios. Next, we demonstrated that Gag bound to USvRNA formed a vRNP that co-localized with Rev in three-dimensional space within a subcompartment of the nucleus. Rev not only enhances gRNA packaging, but was reported to interact with HIV-1 Gag in nucleoli [19,48,70]. USvRNA has also been shown to traffic through nucleoli [71,72], lending support to the idea that vRNPs containing Rev and Gag may pass through nucleoli, potentially interacting with host RNAs or proteins along the way that could contribute to vRNP complex formation, stability, or transport. In addition, Gag was shown to bind to the Rev-responsive element (RRE) at the 3′end of the genome [73], although the functional relevance of the Gag-RRE interaction remains unknown. It is possible that Gag could bind to the RRE sequence, in addition to binding to psi, to form an export competent vRNP complex with Rev, although this vRNA complex does not appear to depend primarily on CRM1 for export based on our results.

LMB does not directly affect transcription [74,75,76], but prevents CRM1 mediated nuclear export of the USvRNA by Rev; therefore, we expected to see an increase in nuclear USvRNA foci following LMB treatment (Figure 6). To our surprise, we observed a modest, yet significant increase in the number of nuclear Gag foci. Previous reports indicated that HIV-1 Gag does not undergo CRM1 mediated export [18,21], although the analysis performed in prior studies was different from the current study and may have missed a modest increase. Even though LMB treatment resulted in an increased number of nuclear HIV-1 Gag-GFP and USvRNA foci, there was no significant change in co-localization. Therefore, Gag-USvRNA co-localization does not appear to be driven by simple mass action, suggesting instead that Gag-USvRNA co-localization occurs prior to formation of the CRM1-nuclear export complex. It is possible that there is a window of opportunity for HIV-1 Gag to associate with the USvRNA in early transcription and if the window closes, HIV-1 Gag does not appear to be able to associate with the USvRNA no matter how much Gag or USvRNA is present in the nucleus. Taken together, these results suggest that nuclear egress of the portion of HIV-1 Gag that comprises the vRNP is dependent on export of USvRNA through the CRM1-dependent Rev pathway, which has been linked to efficient gRNA packaging [70,77,78]. Considering that Rev binds HIV-1 USvRNA co-transcriptionally [48,49] and the cytoplasmic fate of RNAs can be affected by co-transcriptional events [79,80] it is possible that Gag and Rev bind to newly transcribed USvRNA to “mark” it as gRNA for packaging into virions.

It is not clear why a subset of Gag and USvRNA molecules were present alone in the nucleus rather together in a vRNP complex (e.g., co-localization of Gag with USvRNA was 39% and co-localization of USvRNA with Gag was 20%). One explanation is that there could be multiple functions of USvRNA and Gag in the nucleus. The USvRNA is used as both mRNA and gRNA [81], therefore it is reasonable that only a portion of the total USvRNA pool (i.e., the gRNA) is associated with nuclear Gag. As mentioned above, it is possible that Gag binding to USvRNA during transcription identifies it as gRNA, similar to the co-transcriptional binding of factors that determine the cytoplasmic fates of cellular RNAs [82,83]. Furthermore, recent studies by Chen et al. support the idea that HIV-1 Gag selectively binds non-translating USvRNA for packaging into virions, and that the level of Gag expression does not change the ratio of USvRNA in the cytoplasm used for as mRNA versus gRNA [84]. Another possible explanation for the relatively low co-localization of nuclear Gag-GFP or CFP with USvRNA is the detection limit of fluorescent microscopy, which may not be sensitive enough to visualize small nuclear Gag foci. In future studies, the use of single molecule tags to label Gag, such as Halo or SNAP-tag, along with live-cell super-resolution imaging techniques may allow better visualization of vRNP biogenesis and trafficking from the site of transcription to the plasma membrane.

Although these current data do not definitively show that association of nuclear HIV-1 Gag and USvRNA are required for gRNA packaging, previous findings need to be considered in the context of our study. Grewe et al. reported that HIV-1 Gag is unable to export USvRNA from the nucleus and promote efficient gRNA packaging; however, those experiments were all performed in the absence of Rev [18]. In contrast, our experiments were conducted using proviruses that were Rev-dependent, and furthermore, we provided evidence for three-way co-localization of Rev, Gag, and USvRNA in the nucleus. Whereas our data suggest that the initial association between HIV-1 Gag and USvRNA may occur co-transcriptionally, it is also important to consider that the initial binding of HIV-1 gRNA and Gag could occur in both the nucleus and the cytoplasm, which would not be mutually exclusive. Studies from the Lingappa laboratory suggest that vRNPs in cytoplasmic RNA granules associated with host factors DDX6 and ABCE1 [6] may be assembly precursors, and the Mouland laboratory has shown that Staufen is associated with HIV-1 gRNA [8]. Thus, further studies will be needed to determine whether these cytoplasmic complexes described by others are related to the nuclear HIV-1 RNPs we observed.

It is intriguing to consider that HIV-1 Gag may play a variety of roles in the nucleus, such as modulating cellular or proviral transcription, splicing, nuclear organization, or chromatin modification, which may be mediated by HIV-1 Gag foci that remain unassociated with USvRNA. A variety of host proteins involved in nuclear processes were found to interact with HIV-1 Gag in multiple studies using mass spectrometry [65,66,67,68,69], indicating that this line of investigation would expand our understanding of Gag functions in the nucleus. Furthermore, it is plausible that host nuclear proteins may recruit Gag to sites of USvRNA transcription at the proviral integration site in T cells reactivated from latency. Given our observation that HIV-1 Gag is localized at or near transcription sites in association with USvRNA, the effects of HIV-1 Gag on splicing and transcription will be important to examine. Regardless of the function(s) of nuclear HIV-1 Gag that are ultimately discovered, the observations presented in this report warrant greater exploration.

## Supplementary Materials

The following are available online at https://www.mdpi.com/1999-4915/12/11/1281/s1, Figure S1: Transcription inhibition decreases the number of nuclear HIV-1 Gag and USvRNA foci, Figure S2: Treatment of cells with LMB causes an increase in the number of HIV-1 Gag nuclear foci, Video S1: HIV-1 Gag (green) co-localizes with USvRNA (red) within the nucleus and cytoplasm, Video S2: HIV-1 vRNPs (white) are present within the nucleus and cytoplasm, Video S3: HIV-1 Gag-GFP (green) co-localizes with USvRNA (red) in three-dimensions.
