# Supplementary material for: HIV-1 Gag Forms Ribonucleoprotein Complexes with Unspliced Viral RNA at Transcription Sites
Source: Viruses. 2020 Nov 9;12(11):1281. doi: 10.3390/v12111281 (PMC7696413; doi:10.3390/v12111281)
Supplement: Supplementary file 1 [file viruses-12-01281-s001.zip › Supplemental Figures and video legends_09.22.2020.docx]

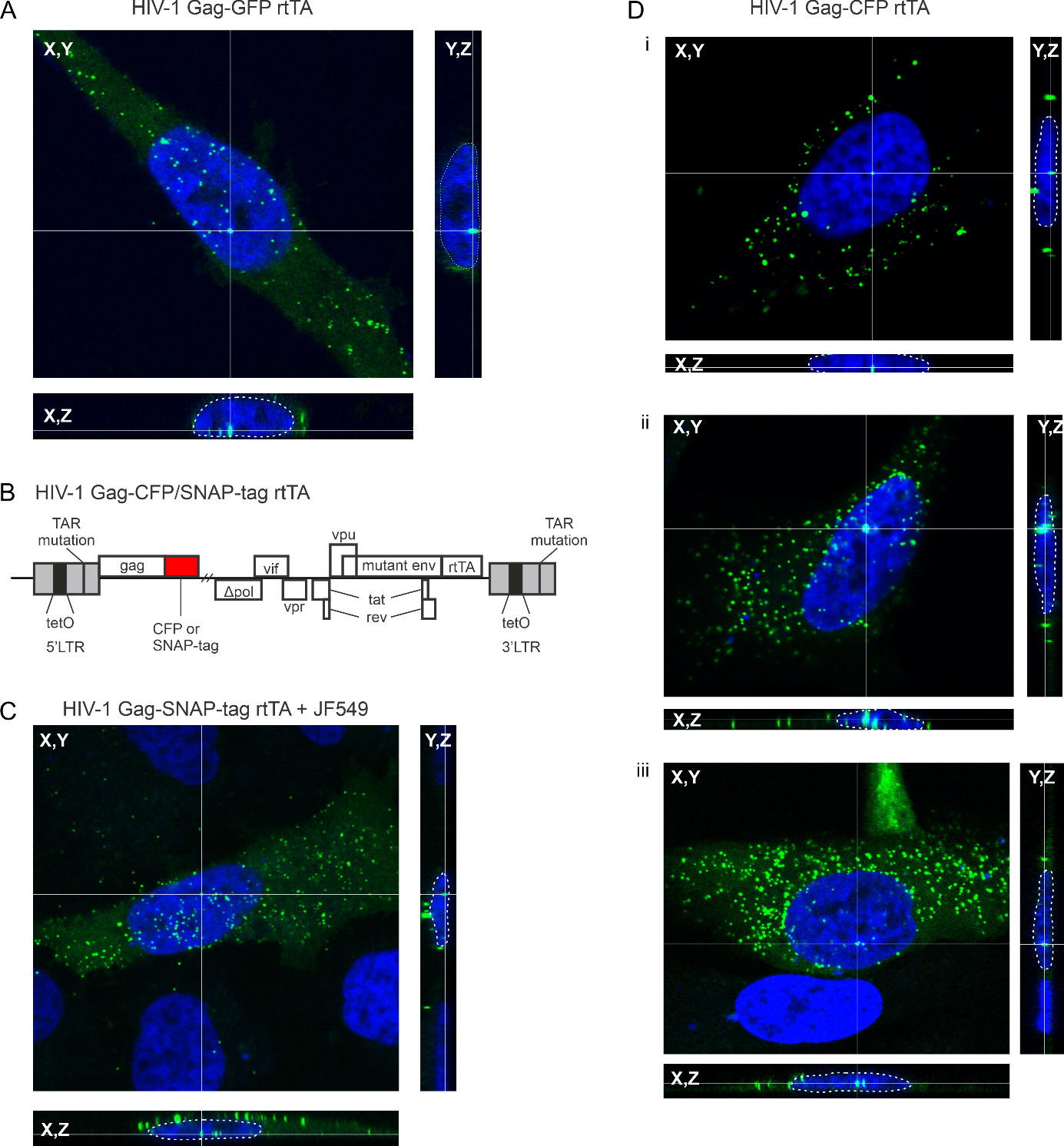
**Figure S1: HIV-1 Gag foci are present in the nucleus of different cell types regardless of the tag to which Gag is fused.** Multiple cell types were transfected with various Rev-dependent Gag constructs. Gag foci (as indicated by the white crosshairs) is present in the nucleus (DAPI-blue; white outline) regardless of cell type or fluorophore/dye to which it was fused. Confocal z-stacks of cells were used to generate cross-sections to show that Gag is present in the nucleus in three dimensions. **(A)** HIV-1 Gag-GFP is expressed from HeLa cells containing a stably-integrated Dox-inducible provirus (HIV-1 Gag-GFP rtTA). **(B)** Schematic of HIV-1 Gag-CFP/ SNAP-tag rtTA doxycycline-inducible construct that expresses Gag fused to either CFP or SNAP-tag. **(C)** rtTA HeLa cell line transfected with a Dox-inducible provirus that produces Gag-SNAP-tag. The Gag-SNAP-tag was labeled with SNAP-ligand JF549. **(D)** Examples of HIV-1 Gag-CFP rtTA co-expressed with pPB-t-rtTA in multiple cell types. (i) HeLa cell with transfected with a Dox-inducible HIV-1 Gag-CFP rtTA provirus that expressed Gag-CFP. (ii and iii) U2OS cells


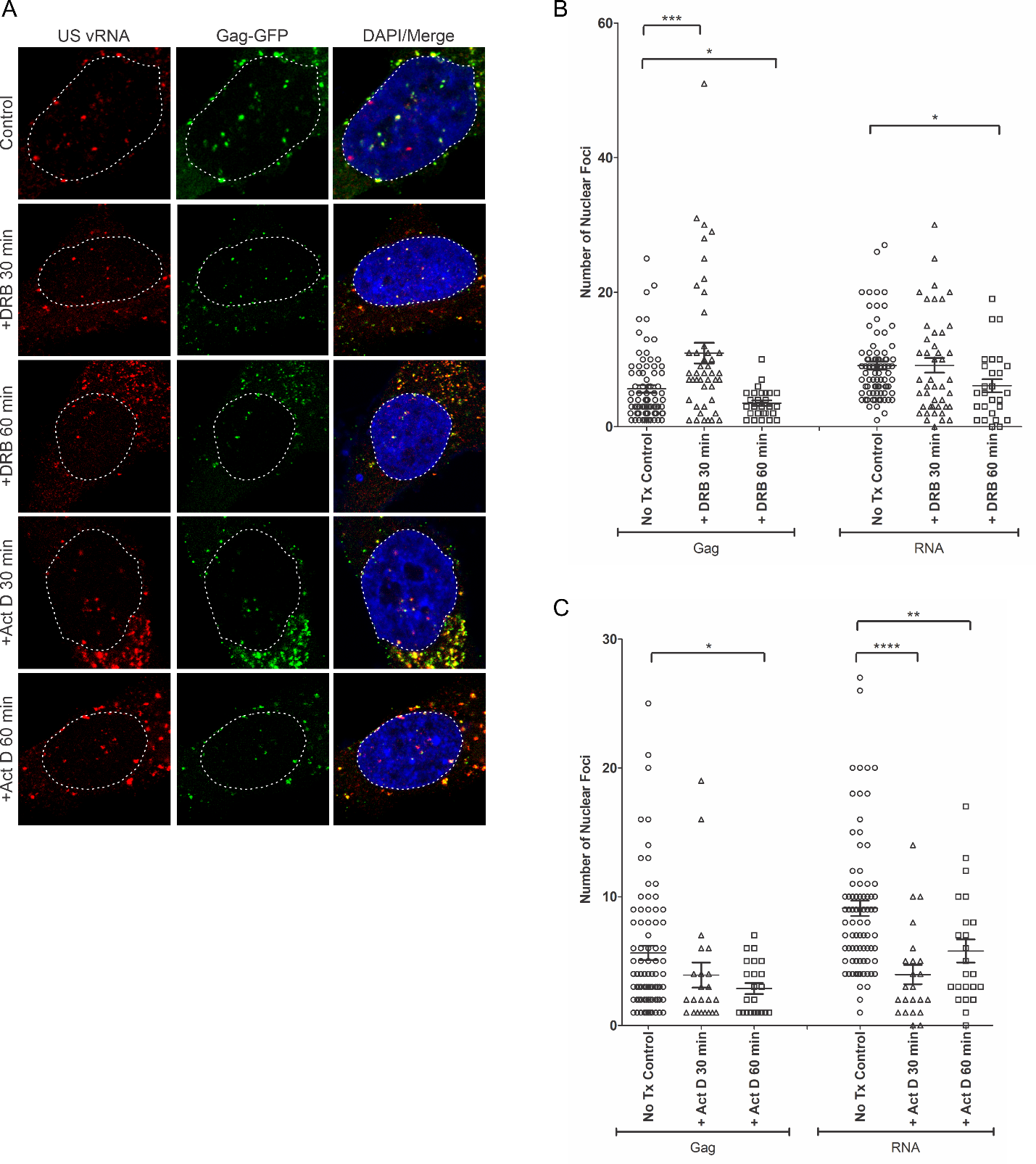


**Figure S2: Transcription inhibition decreases the number of nuclear HIV-1 Gag and US-vRNA foci. (A)** Confocal images of single z-slices of the 3D volume renderings presented in Figure 5C. US-vRNA (red, detected by smFISH), HIV-1 Gag-GFP (green), and co-localized foci (yellow) within the nucleus (DAPI, blue) following treatment of Dox-induced HIV Gag-GFP rtTA cells with the transcription inhibitors DRB or Act D for 30 or 60 min prior to fixation. **(B)** Quantitative analysis depicting the mean number of HIV-1 Gag-GFP and US-vRNA nuclear foci in Dox-induced HIV-1 Gag-GFP rtTA cells following transcription inhibition with DRB (top) or Act D (bottom) for 30 or 60 min compared to the untreated control cells.


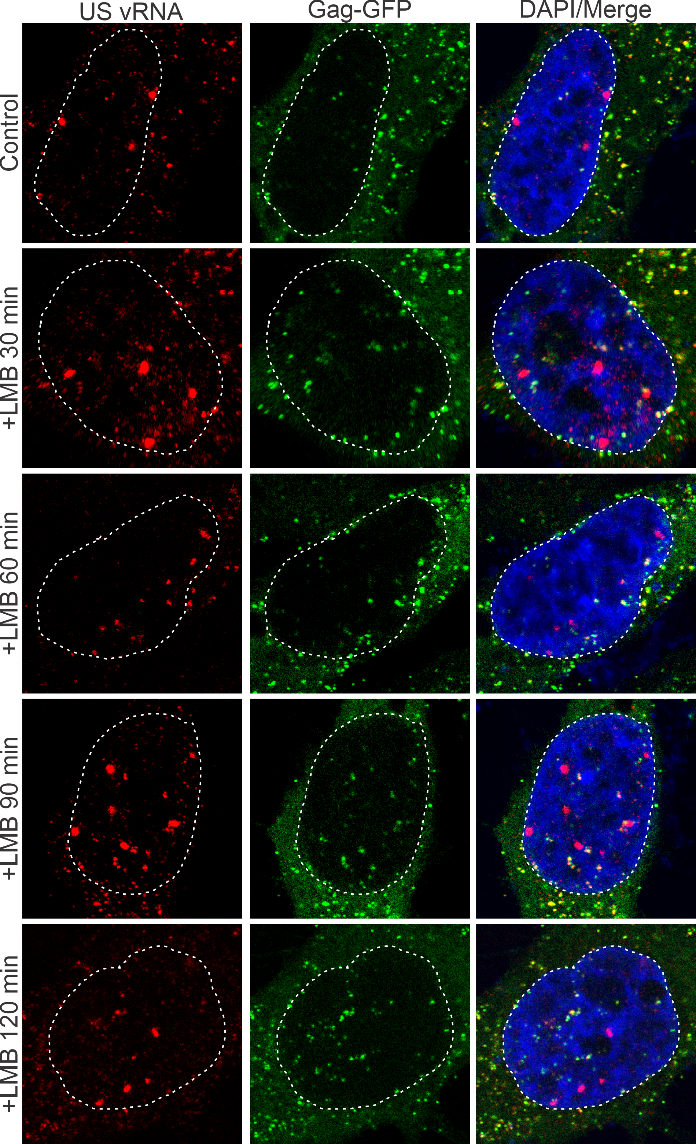


**Figure S3: Treatment of cells with LMB causes an increase in the number of HIV-1 Gag nuclear foci.** Confocal images of single z-slices of the 3D volume renderings presented in Figure 6C. Nuclear localization of HIV-1 Gag-GFP (green), US-vRNA (red, detected by smFISH), and co-localized foci (yellow) within the nucleus (DAPI, blue) following treatment of Dox-induced HIV Gag-GFP rtTA cells with the CRM1 inhibitor LMB for 30, 60, 90, or 120 min prior to fixation.

**Video 1:** **HIV-1 Gag (green) co-localizes with US-vRNA (red) within the nucleus and cytoplasm.** HeLa cells containing a stably-integrated, dox-inducible provirus that expresses Gag-GFP (green) were subjected to smFISH to label US-vRNA (red). Scrolling through each z-plane reveals vRNPs within the nucleus and the cytoplasm.

**Video 2:** **HIV-1 vRNPs (white) are present within the nucleus and cytoplasm.** A white co-localization channel was generated from the cell in Supplemental Movie 1 to highlight the vRNPs. Scrolling through each z-plane reveals vRNPs within the nucleus and the cytoplasm.

**Video 3: HIV-1 Gag-GFP (green) co-localizes with US-vRNA (red) in three-dimensions.** A 3D surface rendering was generated from a HeLa cell containing a stably-integrated, dox-inducible provirus that expresses Gag-GFP and was subjected to smFISH to label US-vRNA. The whole cell is rotated before being bisected in XY by the orthogonal clipping plane to allow visualization of vRNPs within the perichromatin DAPI-poor regions. To highlight vRNP complexes, a surface was generated from a white co-localization channel.
